# Congenital cardiac masses: a case report

**DOI:** 10.1186/s13256-022-03371-1

**Published:** 2022-04-22

**Authors:** Mohammed Alsabri, Alejandro Gonzalez, Aaron Sircy, Sai Sarada Policherla, Kemi Mascoll-Robertson

**Affiliations:** 1 Emergency Department, Al Thawra Modern General Hospital (TMGH), Sana’a, Yemen; 2 Pediatrics, 1 Brookdale University Hospital and Medical center, 1 Brookdale Plaza, Brooklyn, NY 11212 USA; 3 Saba University School of Medicine, The Bottom, Caribbean The Netherlands

**Keywords:** Tuberous sclerosis, Rhabdomyoma, Cardiac tumor

## Abstract

**Background:**

Cardiac tumors in infants and children are rare. The most common cardiac tumor is rhabdomyoma, which may be associated with tuberous sclerosis. However, not all cardiac rhabdomyomas are pathognomonic for tuberous sclerosis, and not all congenital cardiac tumors are rhabdomyomas. During the prenatal period, early cardiac tumor detection provides important information about fetal wellbeing, delivery planning, and necessary postnatal care.

**Case presentation:**

We report a 36-year-old African American pregnant women. At 32 weeks 5 days gestational age, the male fetus had a fetal echocardiogram due to fetal arrhythmia. The fetal echocardiogram showed two small echogenic, RV apex and septal masses, suspicious of rhabdomyomas. After a routine pregnancy and a normal spontaneous vaginal delivery (39 weeks 1 day), the male baby was admitted to the neonatal intensive care unit for further monitoring and postnatal evaluation.

**Conclusions:**

Rhabdomyomas are extremely rare and unique tumors. These tumors are very dangerous, but they usually regress after birth. During the prenatal period, early cardiac tumor detection provides important information about fetal wellbeing, delivery planning, and necessary postnatal care. We present this case to share our findings with our pediatric colleagues. Although a rarely reported case, we hope this cardiac rhabdomyoma case report and literature review can increase cardiac tumor awareness.

## Introduction

Pediatric heart tumors are extremely rare. However, individual institutions have published many unique case studies. Primarily, these case studies discuss cardiac rhabdomyomas, the most common fetal cardiac tumor [1]. Of note, the tumor incidence is very low (1/40,000), and these rhabdomyomas can be found with routine prenatal ultrasound as early as the 15–30th gestational weeks [1]. Studies have shown that 60%–90% of cardiac rhabdomyomas are associated with tuberous sclerosis [2]. However, not all cardiac rhabdomyomas indicate tuberous sclerosis. Tuberous sclerosis is an autosomal dominant disease with varied clinical manifestations [2]. Of cases, 30% are inherited while 70% are de novo mutations. The de novo mutations inactivate *TSC1* (9q34.3) and *TSC2* (16p13.3) [1]. These genes are responsible for the tuberin and hamartin proteins, which are responsible for tumor suppression [1]. Tuberous sclerosis may be associated with cardiac rhabdomyomas. These hamartomas (rhabdomyomas) may present with many unique complications. First, the rhabdomyoma(s) size and location can be problematic. Rhabdomyomas can occupy the interventricular septum, right and left ventricles, or atrioventricular valves [3]. Most pediatric heart tumors are rhabdomyomas, but not every pediatric cardiac tumor is a rhabdomyoma. We present a case study of a newborn term male infant, born at 39 weeks 1 day gestational age (GA). His prenatal echocardiography showed two cardiac tumors, suspicious of rhabdomyomas. After birth, the term male infant was admitted to the neonatal intensive care unit (NICU). Due to the rarity of cardiac tumors, we believe that this case report can help aid pediatric diagnosis and treatment.

## Case presentation

**Fig. 1:**
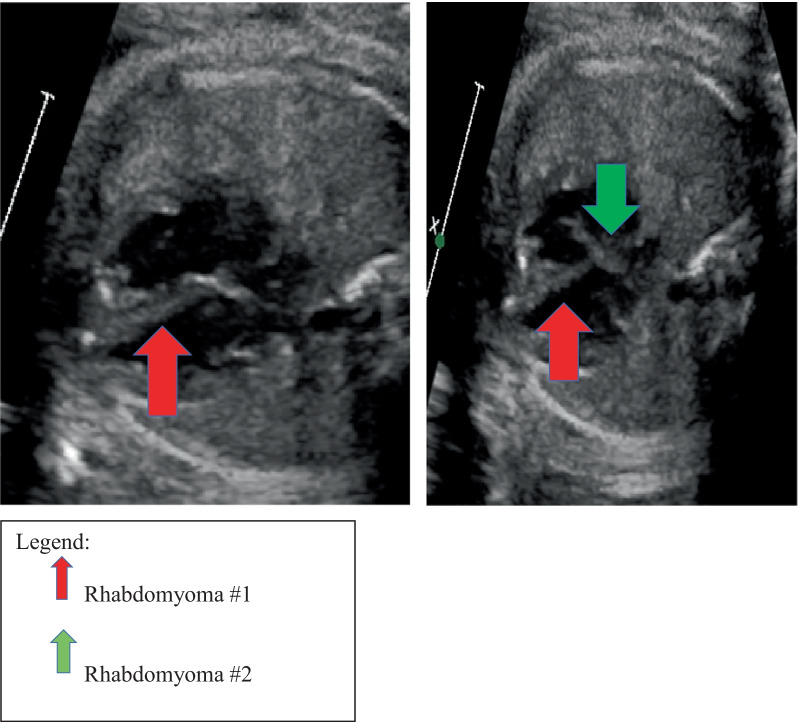
Fetal echocardiography at 32 weeks 5 days gestational age showed two similar small echogenicity in RV apex and septal aspect (4 mm × 2.6 mm) (arrows) suspicious of rhabdomyomas. Normal valvar function

We report the case of a term, African American, male infant delivered by normal spontaneous vaginal delivery (NSVD). Birth weight was 3.03 kg. The baby’s mother had prenatal care × 12 visits. She had a fetal echocardiography done at 32 weeks 5 days gestation, for a fetal arrhythmia. The echo showed two small echogenic, RV apex and septal masses, suspicious of rhabdomyomas (Fig. 1). Next, her third trimester prenatal ultrasound showed oligohydramnios (amniotic fluid index, AFI 4.86). Prior to delivery, the patient’s mother was group B Strep (GBS) positive; she received six doses of intrapartum penicillin. After an uncomplicated birth, the baby was admitted to the NICU for postnatal cardiac evaluation and tuberous sclerosis concerns. In view of the prenatal fetal echocardiogram findings, multiple pediatric specialties (pediatric cardiology, neurology, and pediatric ophthalmology) were consulted and involved in the infant’s care. After admission, the infant had a brown-colored emesis episode, and he was made nothing by mouth (NPO). A gastric lavage was performed, and intravenous fluids were started. No further emesis episodes were observed, and PO (by mouth) feeds were soon started, and gradually increased. The infant tolerated full PO enteral feeds well for the rest of the hospital stay without further concerns. In view of the prenatal fetal echocardiogram findings, an electrocardiogram (EKG) and transthoracic echocardiogram were performed. The EKG showed normal sinus rhythm, and the EKG was normal for age. These masses appeared smaller in the postnatal than on the prenatal echocardiogram evaluation (per the echo report, the cardiologist could not appreciate the dimensions of the tumors). There was no cardiac inflow or outflow obstruction, and normal cardiac structure and function were reported. There were no cardiac rhythm abnormalities. During the 4-day hospital course, the infant remained hemodynamically stable and well. Pediatric cardiology recommended infant reevaluation and imaging in 3–4 months. Imaging could be performed earlier if the size of the cardiac masses [2] caused hemodynamic instability. Next, a pediatric neurology consultation was obtained, reporting an unremarkable examination. The infant did not have any dermatological findings, with no hypopigmented or hyperpigmented lesions. Also, cranial and renal ultrasounds were performed and were unremarkable. A brain magnetic resonance imaging (MRI) was performed, and the patient did not have any intracranial manifestations that suggested tuberous sclerosis. Next, genetic testing was performed (tuberous sclerosis panel), and a pediatric genetic appointment was scheduled in 1–2 months. Pediatric ophthalmology was consulted to rule out tuberous sclerosis ocular involvement. Pediatric ophthalmology reported no abnormalities, well-formed eyes, bilateral anterior segment within normal limits, bilateral D/M/V (disc, macula, vessels) within normal limits, and zone 3 retinal vascularization. Pediatric ophthalmology recommended follow-up in 1 month. Throughout the entire 4-day NICU course, the infant remained on room air, and he maintained adequate oxygen saturation. He had stable vitals with no tachycardia, bradycardia, arrhythmia, or hypotensive episodes. He was active and was easily aroused. He had symmetric and adequate upper/lower extremity tone, and he had appropriate deep tendon reflexes since admission. No abnormal movements or seizure-like activity was observed, and he tolerated PO feeds well. At the time of discharge, outpatient follow-up appointments were arranged with the primary pediatrician, genetics, pediatric cardiology, pediatric neurology, pediatric ophthalmology, and dermatology. A 3-month follow-up report showed that both rhabdomyomas had disappeared. Finally, the tuberous sclerosis genetic tests were negative.

## Discussion

### Infant cardiac tumors and differentials

In infancy, cardiac rhabdomyomas, fibromas, teratomas, and rhabdomyosarcomas are usually rare and benign. During the second and third trimester, echocardiography can diagnose these fetal cardiac tumors [4]. Rhabdomyomas account for more than 60% of infancy and childhood cardiac tumor cases [5]. Rhabdomyomas are muscle cell hamartomas in ventricular and septal walls [2, 5]. In utero, maternal estrogen can enlarge fetal cardiac rhabdomyomas [5]. Rhabdomyomas usually grow up to the 32nd gestational week [6]. Their average diameter is 17 mm [5]. On cardiac ultrasound, cardiac rhabdomyomas are homogeneous, nonvascular, round masses [5]. Pathologically, rhabdomyomas are soft, noncapsulated tumors. One or multiple rhabdomyomas can be found (case dependent). Rhabdomyomas are usually asymptomatic. Depending on size and location, rhabdomyomas can cause arrhythmias, fatal heart cavity obstructions, and rarely intrauterine myocardial infarction due to coronary artery compression [2, 7, 8]. Fetal cardiac tumors greater than 20 mm in diameter pose a higher prenatal death risk [2, 9]. While large or diffuse rhabdomyomas are uncommon, they can cause intracavitary extension [2, 9, 10]. When the tumor(s) obstruct normal heart function, surgery is recommended [2, 9, 10]. Typically, rhabdomyoma cells lose their mitotic potential and gradually regress by early childhood [2, 5–7, 11]. Postnatal echocardiograms, for at least 1 year, track the rhabdomyoma regression phase and ensures no additional infant health threats [7, 9]. Spontaneous tumor regression and apoptotic inducing factors are critical fetal cardiac tumor markers [2, 11]. This can help differentiate benign rhabdomyomas, malignant neoplasms, and Merkel cell skin tumors [11]. To narrow the differential, a prenatal and postnatal echocardiography must show clear tumor growth and regression. The echocardiography can exclude false positives, and identify unique tumor characteristics [2]. Possible cardiac rhabdomyoma differential diagnoses are fibromas or teratomas [5]. Fibromas are the second most common fetal and childhood cardiac tumor [5]. They are usually a large single mass in the interventricular septum or the free wall of the left ventricle [5]. Fibromas grow quickly and can cause severe inflow and outflow complications [5, 7]. Next, teratomas are rarely found within the heart. Teratomas are usually located in the pericardial cavity near the aorta and the pulmonary artery [5, 7]. They have a cystic tumor nature and contain a variety of immature tissues, with possible malignant differentiation [5, 7]. Fibromas or teratomas do not regress, and surgical removal is recommended. Surgery prevents dangerous cardiovascular hemodynamics and/or arrhythmias [5, 7]. Finally, rhabdomyosarcomas are an extremely rare rhabdomyoma mutation. Rhabdomyosarcomas have similar markers versus rhabdomyomas, but very poor prognosis [5, 12]. Due to their dangerous potential, childhood rhabdomyomas and rhabdomyosarcomas are followed until tumor regression is observed [2].

### Tuberous sclerosis

As stated above, rhabdomyomas are associated with tuberous sclerosis complex (TSC) [2]. TSC is a very rare genetic disease that can be identified by several unique findings. Typically, TSC has the Vogt triad (mental retardation, epilepsy, and facial angiofibromas). However, 50% of patients have normal intelligence, and 25% do not have seizures [13]. TSC’s main cardiovascular feature is a cardiac rhabdomyoma [7]. Rhabdomyomas account for 60–86% of cardiac tumors [9]. However, as stated above, not every cardiac rhabdomyoma equals tuberous sclerosis. Next, patients typically have hypomelanotic macules (90%), facial angiofibromas (75%), and shagreen patches (20–30%) [13]. Finally, a definite TSC diagnosis is made when two major criteria or one major plus two minor features are demonstrated [14]. Our patient had one major tuberous sclerosis criterion (two cardiac rhabdomyomas) and no minor tuberous sclerosis criterion. He had no subependymal nodules, dermatological findings, ocular findings, oral findings, renal findings, or cranial findings. Moreover, he had no seizure activity (during hospital stay or 3 month follow-up), negative brain MRI, negative EKG, and negative tuberous sclerosis genetic test. Due to the patient’s age, mental capacity could not be evaluated. Also, the baby had no tuberous sclerosis family history (per the family). At this time, the baby did not meet the tuberous sclerosis diagnosis criteria.

### Diagnosis

Fetal echocardiogram (32 weeks 5 days), prompted by arrhythmia, showed two small echogenic R apex and septal masses, suspicious of rhabdomyomas. The small cardiac tumors did not compromise any cardiac structures and did not cause any inflow or outflow obstructions. Furthermore, both cardiac tumor diameter measurements were 4.0 mm × 2.6 mm each (prenatal echocardiography, shortly before birth). Due to their rare appearance, cardiac rhabdomyosarcomas, myxomas, and hemangiomas were ruled as possible differential diagnoses. Cardiac teratoma was ruled out because the mass was not cystic or in the pericardial cavity (per echo report) [19]. Cardiac fibroma was ruled out because the cardiac masses did not grow quickly. Per the health records and inpatient work-up, the baby did not have malignant neoplasm, lymphoma, or a Merkel cell skin tumor. As a result, those tumors were eliminated from the differential diagnosis. As seen in the previous section, our patient did not meet the tuberous sclerosis criteria. As a result, tuberous sclerosis is an unlikely differential diagnosis. Due to tumor number, appearance, location, and size regression, the two cardiac tumors were diagnosed as cardiac rhabdomyomas. A 3-month patient follow-up showed that the two small, cardiac rhabdomyomas had completely and spontaneously disappeared.

### Other TSC case studies and literature comparisons

Our research group compared other TSC case studies with our baby’s case. First, many TSC case reports have been completed, but many were not infants [13–15]. TSC patients have been reported at 4, 11, and 26 years of age. Patients were diagnosed after unexplained seizures, skin lesions, or unexplained mental retardation [14–16]. Further medical work-up was completed, and patients had the required two major criteria or one major criteria plus two minor criteria. These cases were treated without any pathological samples, and they were referred to the respective specialties [13–16]. Pathological samples are very helpful, but not a requirement. As mentioned above and seen in the medical literature, TSC diagnosis uses the TSC major and minor criteria, denaturing high-performance liquid chromatography (DHPLC), and direct sequencing [9]. Unfortunately, TSC pathological samples are used from deceased babies. These babies usually have large cardiac tumors, hydrops fetalis, fetal dysrhythmia, or hemodynamic compromise [9]. These pathological evaluations are usually post mortem, rare, and not a diagnosis requirement. Pathological samples are helpful, but genetic analysis is very helpful too. As stated above, tuberous sclerosis is usually due to *TSC1* and *TSC2* mutations [15]. In neonatal mutation cases, *TSC2* mutations are four times more likely than *TSC1* mutations [15]. However, familial tuberous sclerosis cases have equal *TSC1* and *TSC2* mutation rates. Typically, *TSC1* gene mutations are point mutations, whereas *TSC2* mutations lack large sequence fragments. Clinically, *TSC2* mutations are worse than *TSC1* mutations. *TSC2* mutations correspond to worse cognitive deficiencies, and younger age at seizure onset [15]. With these facts, genetic testing can greatly aid clinicians. New medical treatment has shown cardiac rhabdomyoma improvement [17, 18]. As stated above, most cardiac rhabdomyomas shrink over time and, the cardiac tumor mechanism is not perfectly clear. However, not every patient has post-birth tumor size reduction. Li *et al.* [17] reported two patients that had life-threatening problems, and they were not surgical candidates. They were given everolimus (a rapamycin inhibitor) therapy of 2 × 0.5 mg twice a week for 3 months [14]. Both patients had their rhabdomyomas size reduce and clinical status improve. As these circumstances are exceedingly rare, research will evaluate this therapy in the future [17, 18]. Next, medical literature has reported a similar rhabdomyoma case in comparison with our baby’s case. Karatas *et al.* [2] reported an infant with a benign cardiac rhabdomyoma. The benign rhabdomyoma regressed, and the patient did not develop TSC. Moreover, the case report did not reference any hemodynamic instability, seizures, or rashes. This case is almost identical to our patient. Karatas *et al.* [2] followed the same medical management as our baby’s medical management. Karatas *et al.* [2] and our case highlight that not every cardiac rhabdomyoma indicates tuberous sclerosis, and infant follow-up should observe cardiac rhabdomyoma tumor regression.

## Conclusion

Rhabdomyomas are extremely rare and unique tumors. These tumors are very dangerous, but they usually regress after birth. As shown above, these tumors can affect many different organ systems. Therefore, a multi-medical disciplinary approach is a necessity. At the 3-month follow-up, the patient’s cardiac rhabdomyoma masses exhibited size reduction. Cardiac rhabdomyomas have been reported before, but we hope to increase awareness of this rare disease.

## Data Availability

All data are included in the medical record of the patient. Clinical data are available from the corresponding author but only on reasonable request.

## References

[1] Ekmekei E, Ozkan BO, Yildiz MS, Kocakaya B. Prenatal diagnosis of fetal cardiac rhabdomyoma associated with tuberous sclerosis: a case report. Case Rep Women’s Health. 2018;19:e00070. 10.1016/j.crwh.2018.e00070 PMC6071374 30094198

[2] Karatas A, Karatas Z, Ozlu T. Fetal cardiac rhabdomyoma without tuberous sclerosis: a case report. Int J Med Sci Public Health. 2013;2:1095–7.

[3] Sarkar S, Siddiqui WJ. Cardiac rhabdomyoma. StatPearls: StatPearls Publishing; 2021. 32809444

[4] Delmo Walter EM, Javier MF, Sander F, Hartmann B, Ekkernkamp A, Hetzer R. Primary cardiac tumors in infants and children: surgical strategy and long-term outcome. Ann Thorac Surg. 2016;102(6):2062–9. 10.1016/j.athoracsur.2016.04.057 27344282

[5] Uzun O, Wilson DG, Vujanic GM, Parsons JM, De Giovanni JV. Cardiac tumours in children. Orphanet J Rare Dis. 2007;2:11. 10.1186/1750-1172-2-11 PMC3225855 17331235

[6] Colosi E, Russo C, Macaluso G, Musone R, Catalano C. Sonographic diagnosis of fetal cardiac rhabdomyomas and cerebral tubers: a case report of prenatal tuberous sclerosis. J Prenatal Med. 2013;7(4):51–5. PMC3931277 24611096

[7] Sarff B, Floyd R, Bildner A, Stormo J, Fisher K. Fetal echocardiographic detection of cardiac tumors: a case report of multiple fetal cardiac rhabdomyomas. J Diagn Med Sonography. 2019;35(5):426–30.

[8] Kwiatkowska J, Ciemny S, Kozłowski D. Giant cardiac tumours in the newborn: an unusual image. Folia Morphol. 2018;77(3):601–3. 10.5603/FM.a2017.0119 29297180

[9] Chao AS, Chao A, Wang TH, Chang YC, Chang YL, Hsieh CC, Lien R, Su WJ. Outcome of antenatally diagnosed cardiac rhabdomyoma: case series and a meta-analysis. Ultrasound Obstet Gynecol. 2008;31(3):289–95. 10.1002/uog.5264 18307215

[10] Weiland MD, Bonello K, Hill KD. Rapid regression of large cardiac rhabdomyomas in neonates after sirolimus therapy. Cardiol Young. 2018;28(3):485–9. 10.1017/S104795111700244X 29233209

[11] Fukasawa Y, Ishikura H, Takada A, Yokoyama S, Imamura M, Yoshiki T, Sato H. Massive apoptosis in infantile myofibromatosis. A putative mechanism of tumor regression. Am J Pathol. 1994;144(3):480–5. PMC1887082 8129033

[12] Schaaf G, Hamdi M, Zwijnenburg D, Lakeman A, Geerts D, Versteeg R, Kool M. Silencing of SPRY1 triggers complete regression of rhabdomyosarcoma tumors carrying a mutated RAS gene. Can Res. 2010;70(2):762–71. 10.1158/0008-5472.CAN-09-2532 20068162

[13] Dzefi-Tettey K, Edzie EK, Gorleku P, Piersson AD, Cudjoe O. Tuberous sclerosis: a case report and review of the literature. Cureus. 2021;13(1):e12481. 10.7759/cureus.12481 PMC7854338 33552794

[14] Shrestha S, Shrestha S, Ojha AR. Case report on tuberous sclerosis. J Kathmandu Med College. 2014;2(4):208–10.

[15] Sarkar S, Khaitan T, Sinha R, Kabiraj A. Tuberous sclerosis complex: a case report. Contemp Clin Dentistry. 2016;7(2):236–9. 10.4103/0976-237X.183071 PMC4906871 27307675

[16] Mongrain V, van Doesburg NH, Rypens F, *et al*. A case report of severe tuberous sclerosis complex detected in utero and linked to a novel duplication in the TSC2 gene. BMC Neurol. 2020;20:324. 10.1186/s12883-020-01905-y PMC7460776 32873234

[17] Li H-F, Wang D, Li J-Q, Zhang L, Zhang X, Qi H-B, Li J-N. Tuberous sclerosis complex secondary to the presence of fetal cardiac rhabdomyoma: a case report and literature review. Maternal-Fetal Medicine. 2020;2(4):240–3.

[18] Behram M, Oğlak SC, Acar Z, Sezer S, Bornaun H, Çorbacıoğlu A, Özdemir İ. Fetal cardiac tumors: prenatal diagnosis, management and prognosis in 18 cases. J Turkish German Gynecol Assoc. 2020;21(4):255–9. 10.4274/jtgga.galenos.2020.2019.0180 PMC7726467 33274615

[19] Bejiqi R, Retkoceri R, Bejiqi H. Prenatally diagnosis and outcome of fetuses with cardiac rhabdomyoma—single centre experience. Open Access Macedonian J Med Sci. 2017;5(2):193–6. 10.3889/oamjms.2017.040 PMC5420773 28507627

